# Mycobacterial identification on homogenised biopsy facilitates the early diagnosis and treatment of laryngeal tuberculosis

**DOI:** 10.1515/med-2020-0171

**Published:** 2020-06-05

**Authors:** Zhenjun Yu, Ruyue Lu, Meifu Gan, Xi Tu, Zebao He

**Affiliations:** Department of Infectious Diseases, Taizhou Enze Medical Center (Group) Enze Hospital, No. 1, Tongyang Road, Taizhou 318000, Zhejiang, China; Department of Clinical Laboratory, Taizhou Enze Medical Center (Group) Enze Hospital, No. 1, Tongyang Road, Taizhou 318000, Zhejiang, China; Department of Pathology, Taizhou Enze Medical Center (Group) Enze Hospital, No. 1, Tongyang Road, Taizhou 318000, Zhejiang, China

**Keywords:** laryngeal tuberculosis, mycobacterial identification, gene chips

## Abstract

**Introduction:**

The incidence of laryngeal tuberculosis has increased gradually in recent years. Laryngeal tuberculosis has strong infectivity and atypical clinical manifestations. Hence, establishing the early diagnosis of laryngeal tuberculosis is considered difficult, resulting in the high rate of misdiagnosis of laryngeal tuberculosis and increased rates of tuberculosis infection.

**Objective:**

This study aimed to describe a case of laryngeal tuberculosis detected using the mycobacteria gene chips technology, facilitating the early diagnosis and the treatment of laryngeal tuberculosis.

**Case presentation:**

A 27-year-old woman presented with a 7-day history of hoarseness, with a normal routine blood chemistry test and chest computed tomography results. Histological analysis of the vocal cord biopsy showed granulomatous inflammation and the negative acid-fast stain test. The mycobacteria gene chips method was used to directly examine the vocal cord tissue treated with homogenate, and the *Mycobacterium tuberculosis* was successfully identified. Thus, the early diagnosis of laryngeal tuberculosis and the drug sensitivity of rifampin and isoniazid were confirmed. The patient recovered after undergoing a 1-year standard anti-tuberculosis therapy.

**Conclusions:**

Mycobacterial identification on homogenised biopsy using the mycobacteria gene chips method significantly facilitates the early diagnosis and the treatment of tuberculosis.

## 1 Introduction

In recent years, the epidemic status of tuberculosis (TB) has gradually increased along with the human immunodeficiency virus (HIV) infection and an aging population [1,2]. In 2016, an estimated 1.7 million people died of TB globally, with approximately 10.4 million new cases, half of which were concentrated in five countries, including China and India [3]. TB infections in the head and neck have also increased significantly, specifically in developing countries [4]. Laryngeal TB, which accounts for approximately 1% of TB cases, is the most common extrapulmonary TB [5]. Laryngeal TB has strong infectivity and atypical clinical manifestations. Hence, establishing the early diagnosis of laryngeal TB is considered difficult, resulting in the high rate of misdiagnosis of laryngeal TB and increased rates of TB infection.

Early, rapid, and accurate diagnosis of TB is the key to TB control. The most widely used method for TB diagnosis is a simple and inexpensive acid-fast staining. However, the positive rate of this method is low, and it is impossible to distinguish mycobacterium TB (MTB) from non-TB mycobacterium. MTB culture is the gold standard used to establish the diagnosis of active pulmonary TB, but the culture cycle of MTB is long. Roche culture needs to be cultured for at least 4–8 weeks, but liquid culture, such as BACTEC-MGIT 960 system, takes less time to be cultured (1–2 weeks). Hence, the clinical demand in establishing the diagnosis of laryngeal TB is considered difficult [6]. Currently, the gene chips technology has been widely used in TB diagnosis. The microarray obtained the sample information by detecting the signal strength of hybridisation between a variety of probes fixed on a special carrier and the nucleic acid of the sample to be tested. Since the 16S rRNA and rpoB genes of MTB are highly conserved sequences with species-specific characteristics, mycobacterium can be directly identified as a species [7]. In this study, we will introduce a case of laryngeal TB that was diagnosed early with the application of MTB gene chips technology, suggesting that a good treatment effect was finally obtained, facilitating the diagnosis and the treatment of laryngeal TB.

## 2 Case presentation

**Figure 1:**
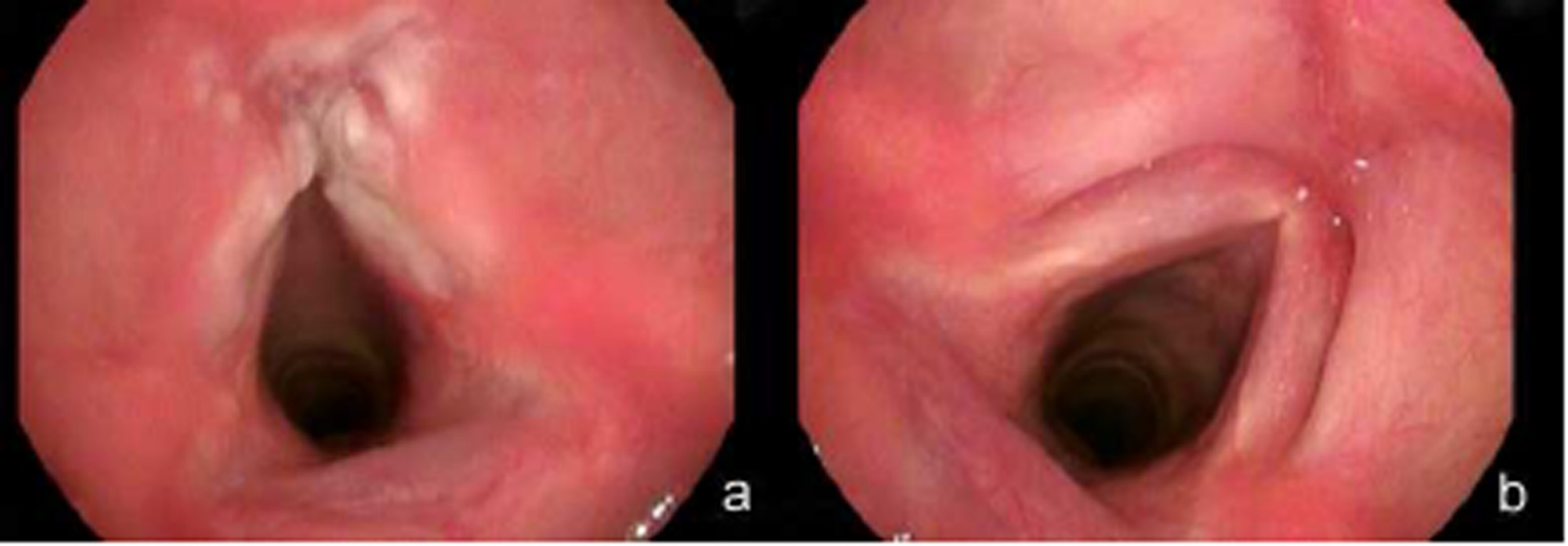
(a) Laryngoscopy revealed laryngeal mucosa hyperaemia and milky hyperplastic lesion in the bilateral vocal cords. (b) Laryngoscopy revealed the laryngeal mucosa without hyperaemia, and the hyperplastic lesion in the vocal cords completely disappeared after the patient underwent a 1-year standard anti-tuberculosis therapy.

**Figure 2:**
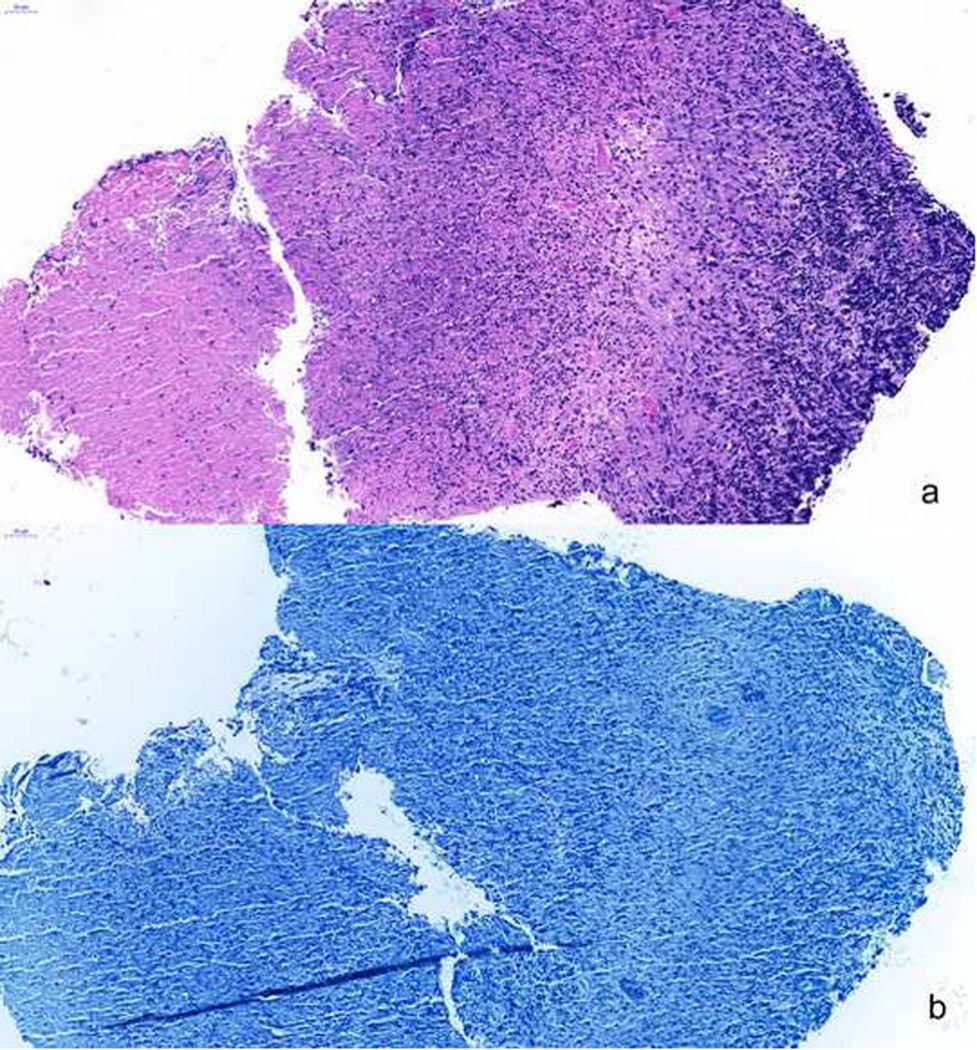
(a) Original magnification ×200. Histological analysis of the biopsy specimen from the vocal cords revealed granulomas with inflammatory cell infiltration in the squamous epithelium and interstitial tissue. (b) The result of the acid-fast stain for vocal cord biopsy was negative.

The patient was a 27-year-old Chinese woman who was a laboratory doctor in a hospital involved in the treatment of sputum samples from patients with TB. The patient had never received immunosuppressive treatment, was serologically negative for HIV and hepatitis, and had no family history of TB. She was admitted to a hospital following a 7-day history of hoarseness without fever, cough, expectoration, emaciation, sweating, sore throat, or other non-specific symptoms such as anorexia, fatigue, and muscle aches. The patient’s chest computed tomography (CT) and routine blood chemistry results, erythrocyte sedimentation rate (ESR), and tumour markers were all normal. The results of the acid-fast stain test of sputum smear and mycobacterial identification on throat secretion by the polymerase chain reaction (PCR) were all negative. The laryngoscopy revealed laryngeal mucosa hyperaemia and milky hyperplastic lesion in the bilateral vocal cords (Figure 1a). Histological analysis of the biopsy specimen from the vocal cords revealed granulomas with inflammatory cell infiltration in the squamous epithelium and interstitial tissue (Figure 2a) and negative results of periodic acid-Schiff stain (PAS), acid-fast stain (Figure 2b), and Gomori methenamine silver stain (GMS).

**Figure 3:**
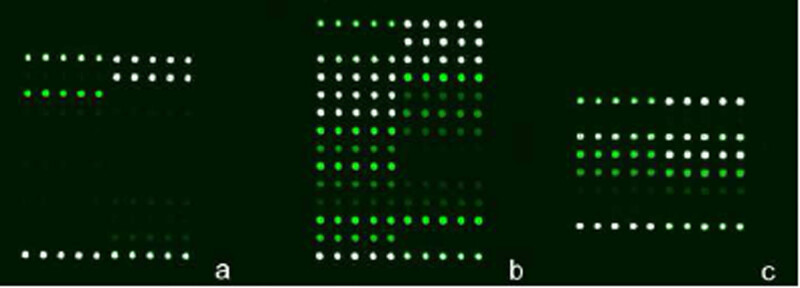
(a) Identification of bacteria by mycobacteria gene chips showed *Mycobacterium tuberculosis*. (b) Identification of rifampin-resistant gene detected by mycobacteria gene chips showed that 511WT, 513WT, 516WT, 526WT, 531WT, and 533WT were all wild type. (c) Identification of isoniazid-resistant gene detected by mycobacteria gene chips showed that katG315 and inhA-15 were wild types.

We cut the biopsy vocal cords directly, digested the homogenised tissues with proteinase K, and prepared template deoxyribonucleic acid (DNA) for PCR amplification. The mycobacteria gene chips (Mycobacteria Identification Array Kit and Tuberculosis Drug Resistance Detection Array Kit, CapitalBio Corp., China) were used to identify the bacteria and resistance genes. The results showed that MTB was positive (Figure 3a), and the detection of resistance genes indicated that rifampin and isoniazid had a wild-type genotype (Figure 3b and c).

Consequently, the patient was diagnosed with laryngeal TB and was treated with an antibiotic regimen combining rifampicin (10 mg/kg/day), isoniazid (5 mg/kg/day), pyrazinamide (30 mg/kg/day), and ethambutol (20 mg/kg/day) for 3 months and subsequently rifampicin (10 mg/kg/day), isoniazid (5 mg/kg/day), and ethambutol (20 mg/kg/day) for 9 months. After 1 year of well-tolerated treatment, the patient recovered, and the hyperplastic lesion in the vocal cords completely disappeared under laryngoscopy (Figure 1b).

The authors received research approval from Institutional Medical Ethic Review Board.

Informed consent was obtained from the patient included in this study.

## 3 Discussion

Laryngeal TB can be divided into primary and secondary laryngeal TB. Primary laryngeal TB is diagnosed when only the throat is infected with tubercle bacillus and no other active TB lesions are present. Secondary laryngeal TB is always secondary to active TB of other organs, specifically the pulmonary TB [8]. The larynx is rarely the primary site involved and is usually infected via the expectoration of sputum from the tracheobronchial tree, hematogenous spread from other sites, or less frequently via lymphatic spread [9]. Patients with laryngeal TB usually present with laryngeal symptoms and lack typical TB poisoning symptoms [10]. A retrospective study showed that dysphonia and weight loss were the most common manifestations in laryngeal TB. Moreover, 86% of patients had underlying pulmonary involvement, with a 3% mortality rate [11]. The final diagnosis of laryngeal TB requires histopathological examination, granulomas with giant cells with or without caseous necrosis, detection of the tubercle bacillus, and the positive results of acid-fast stain or tubercle bacillus culture [12]. Mycobacterial smear and culture are difficult in these laryngeal locations due to the low concentrations of the bacilli. Therefore, establishing the early diagnosis of laryngeal TB is significantly difficult. Kurokawa et al. [13] finished a retrospective analysis of 1,660 inpatients with TB treated at the Osaka Prefectural Medical Center for Respiratory and Allergic Diseases. Seventeen patients were diagnosed with laryngeal TB. The criterion used to establish the diagnosis was the clinical response of granuloma to anti-TB therapy (15 patients) considering the lack of exact diagnostic evidence.

In this case, the patient had a history of direct contact with the tubercle bacillus. The only symptom was hoarseness for 7 days, and the histological analysis of vocal cord biopsy revealed granulomatous inflammation and a negative acid-fast stain test. Although we highly suspected primary laryngeal TB, there was no specific diagnostic evidence supporting our hypothesis. More importantly, the patient was already highly likely to be infected with drug-resistant MTB or non-tuberculous mycobacteria, such as *Mycobacterium intracellulare* and *Mycobacterium avium*, since she had long been exposed to sputum specimens from patients with various pulmonary TB.

Determining the diagnostic treatment for TB was also significantly difficult because conventional therapies were ineffective for drug-resistant MTB or non-tuberculous mycobacteria [14]. A culture from the vocal cord tissue might assist in the diagnosis despite the prior low success rate. However, the long culture cycle, requiring 1–2 months, could aggravate the condition or lead to permanent damage to the glottis from delayed treatment. We cut the biopsy vocal cord and homogenised it to further determine the mycobacteria by PCR and mycobacteria gene chips. We were able to successfully diagnose laryngeal TB and identify a sensitive therapeutic regimen in a short time.

Previous studies show that the interferon-γ release assay [15], the analysis of bronchoalveolar lavage, the laryngeal biopsy culture, and the stool culture can assist in the diagnosis of laryngeal TB. Matsuura and Yamaji [16] reported a 93-year-old woman with a 2-month history of hoarseness and cough. Chest CT revealed a lesion suggestive of TB in the apex, and the laryngoscopy revealed an ulcerated and granular lesion in the ventricular folds, larynx vestibule, and bilateral vocal cords. Ziehl–Neelsen staining of the bronchoalveolar lavage fluid revealed acid-fast bacilli. Jurado et al. [17] reported a 56-year-old man who presented with a 6-month history of dysphonia. Vocal cord histological examination showed granulomas, giant cells, fibrosis, and necrosis. Cultures from the vocal cord tissue were positive for mycobacterial infection. The last case was a 41-year-old French man with a 3-month history of progressive dysphonia. A laryngoscopic examination revealed an ulcerated lesion of the left vocal cord. Stool cultures collected at days 2 and 3 grew after 29 days of culture on MTB; however, sputum and bronchoalveolar lavage cultures remained negative at 45 days [18]. However, in our opinion, establishing the early diagnosis of laryngeal TB, specifically the primary laryngeal TB, using the mycobacterial smear and culture is considered difficult due to the low concentrations of bacilli in the laryngeal locations, bronchoalveolar lavage, and stools.

In summary, mycobacterial identification on homogenised biopsy using gene chips technology is considered beneficial in establishing the early diagnosis and the treatment of TB. The diagnosis of various TB such as pulmonary, intestinal, renal, bladder, and bone TB may benefit from this method. Considering the early identification of laryngeal TB, appropriate management and treatment of this disease can be facilitated, reducing the prevalence of TB and improving public health [16].

## Abbreviations

CT: computed tomography
DNA: deoxyribonucleic acid
ESR: erythrocyte sedimentation rate
GMS: Gomori methenamine silver stain
HIV: human immunodeficiency virus
PAS: periodic acid-Schiff stain
PCR: polymerase chain reaction
TB: tuberculosis
